# Principles of a New Protocol for Prediction of Azole Resistance in *Candida albicans* Infections on the Basis of *ERG11* Polymorphisms

**DOI:** 10.1007/s00284-016-1039-3

**Published:** 2016-04-23

**Authors:** Monika Caban, Dominik Strapagiel, Jarosław Dziadek, Małgorzata Korycka-Machała, Agnieszka Grzelak

**Affiliations:** Cytometry Lab, Department of Molecular Biophysics, Faculty of Biology and Environmental Protection, University of Łódź, Pomorska 141/143, 90-236 Łódź, Poland; Biobank Lab, Department of Molecular Biophysics, Faculty of Biology and Environmental Protection, University of Łódź, Pilarskiego 14/16, 90-231 Łódź, Poland; Institute of Medical Biology, Polish Academy of Sciences, Lodowa 106, 93-232 Łódź, Poland

## Abstract

In recent years, *Candida albicans* infections treatment has become a growing problem because, among others, pathogenic strains are capable to develop resistance to the administered drugs. The elaboration of rapid and accurate method of resistance assessment is an important goal of many studies. They aim to avoid inappropriate dosage or drug choice, which may be life threatening in case of severe candidiasis. Here we propose a new protocol to predict *C. albicans* infections. The resistance prediction is based on high-resolution melt (HRM) analysis of *ERG11* gene, especially, at the particularly unstable regions. Two statistically significant nucleotide polymorphisms were detected among twenty-seven strains isolated from saliva, one of which was silent mutation (Glu266Asp, Leu480Leu). We propose also HRM analysis as a convenient, simple and inexpensive method of preliminary selection of *C. albicans* DNA samples that vary in *ERG11* nucleotide sequence within presumed region. Taken together, our study provides firm basis for the development of fast, simple and reliable methodology for diagnosis of *C. albicans* infections.

## Introduction

*Candida* spp are opportunistic yeasts that normally inhabit human skin, genitourinary and gastrointestinal tracts. Under some conditions, *Candida* may overgrow and colonize mucosal and skin surfaces [5], which usually leads to easily treated infections. However, in patients with impaired immune system, severe candidiasis and life-threatening systemic infections can develop. Among *Candida* species, *Candida albicans* is the most frequent cause of human infections.

Several different mechanisms of drug resistance have been already described [4, 40]. Resistant *Candida* strains may overexpress membrane transporters which are responsible for drug removal from the cell [25, 28]. Efflux pumps can also be more active in drug-resistant strains than in susceptible ones [38]. Most commonly administered antifungal drugs are azole compounds and the main azole drugs target is lanosterol-14-α-demethylase, encoded by *ERG11*. Point mutations in *ERG11*, as well as overexpression of this gene, are usually involved in the development of resistance to azole-based drugs [6]. Mutations that lead to amino acid substitution may cause changes in the affinity of the enzyme for azole derivatives [6, 30, 41]. Thus, huge effort has been made to develop fast and reliable methods for detecting strains that bear mutations [1, 35, 39]. Most of these methods involve nucleic acid amplification and gel electrophoresis [35].

Among numerous *ERG11* mutations, both homozygous and heterozygous nucleotide changes were reported to correlate with azole susceptibility [6, 29, 41, 42]. A huge progress in *ERG11* mutations identification has been made in recent years and it is well known now, which of them are the most frequent and which are most probably involved in azole resistance [24]. However, detailed drug-resistant mutation pattern of *ERG11* gene is still desired. We suggest that to achieve this aim, it is also important to consider mutations that do not directly change amino acid composition of the enzyme, but may also correlate with resistant phenotype, and thus may serve as convenient drug resistance markers. Likewise, silent polymorphism in drug-resistance transporter MDR1 gene (C3435T) was established as a risk factor for non-clear cell renal cell carcinoma [34]. It has been also shown that (C3435T) polymorphism may lead to the synthesis of protein product with different structural and functional properties, in spite of unchanged amino acid sequence [12]. Proposed molecular mechanism of this phenomena is the existence of frequent and infrequent codons in mRNA which influence the translation kinetics and temporal separation of folding events during protein synthesis causes conformational and functional differences of final protein [13]. Silent mutations in *ERG11* gene of *C. albicans* occur with threefold higher frequency then amino acid changing mutations [21].

HRM (High-Resolution Melt) analysis is a precise and sensitive method, which can be successfully used for identification of species [20]. This technique allows to discriminate amplicons that differ in single nucleotide (single-nucleotide polymorphisms) as they generate various shapes of melting curves when heated after amplification [15]. Therefore, we propose HRM analysis as a rapid and robust method of preliminary scanning amplicons which allows to minimize the number of samples to be sequenced. Thus, it may contribute essentially to save money, time and labor, especially in case of diagnostic laboratories. Here we applied a recent PCR-based technique, high-resolution melting (HRM) analysis, to determine differences in *ERG11* sequence isolated from various *C. albicans* strains and searched for *ERG11* polymorphisms that are statistically significant in drug-resistance prediction.

## Materials and Methods

### Control Strains and Clinical Isolates

Three strains used as control strains were as follows: Ca11r—*C. albicans* (ATCC 10231) and Ca12r—*C. albicans* (ATCC 60193) and Ca1r—clinical *C. albicans* strain identified by MALDI. Seven clinical isolates came from vaginal candidiasis patients (Ca1V–Ca7V) and have been kindly provided by Dr. Agata Karowicz-Bilińska (Medical University of Lodz). 28 strains (Ca1s–Ca82s) were isolated from the saliva of healthy volunteers (Bioethics Committee agreement no. 6./KBBN-UŁ/II/2014). Clinical strains were isolated on yeast extract–peptone–dextrose (YEPD) agar plates supplemented with chloramphenicol. Then, they were directly identified as *C. albicans* species using chromogenic culture media (chromID^®^*Candida*, bioMérieux), HRM and sequence analysis of ITS2 regions (data not shown). All strains were stored at −70 °C as stocks in 8 % DMSO. Strains were cultured for 24 h at 37 °C in YEPD-agar containing 2 % glucose, 2 % peptone, and 1 % yeast extract.

### Antifungal Susceptibility Testing

The susceptibility of reference and vaginal strains to antifungal drugs was tested according to the modified NCCLS M27-A microdilution procedures. Briefly, 1:1 dilutions of fluconazole (FCA), ketoconazole (KET), miconazole (MIC), itraconazole (ITR), amphotericin B (AMB), and 5 flucytosine (5FC) were prepared in 96-well plates in RPMI medium, frozen and thawed when needed. 24-h yeast culture in YPD medium was washed in RPMI medium and 2.5 × 10^3^ cells mL^−1^ were seeded on a 96-well plate. IC50 was determined spectrophotometrically after 48 h of incubation. To determine the saliva isolates, susceptibility ATB FUNGUS3 (BioMerieux) tests were used according to manufacturer’s instructions.

### DNA Isolation

Genetic material was extracted with modified Harju yeast genomic DNA isolation method [8]. Briefly, liquid culture of yeast was pelleted. Cell pellets were resuspended in 200 μl of lysis buffer [2 % Triton X-100, 1 % SDS, 100 mM NaCl, 10 mM Tris–HCl, 1 mM EDTA (pH 8.0)]. Cell suspension was heat shocked on ice bath followed by 95 °C water bath twice. Samples were then vortexed with chloroform, centrifuged and the aqueous layer was transferred to the ice-cold 96 % ethanol. After 5 min incubation, the probes were centrifuged and the supernatant was removed. DNA was washed with 70 % ethanol, pelleted, air-dried, resuspended in 30 µl of sterile water and stored at −20 °C.

### HRM Analysis

For HRM analysis, the whole *ERG11* gene was divided to 9 regions, aprox. 300 bp each. Primers were designed so as to obtain all specific PCR products at the same annealing temperature. Sequences of primers used for amplification are given in Table 1.

**Table 1:** List of primers used for the amplification in HRM analysis

	PCR product size (bp)	Sequence of forward primer	Sequence of reverse primer
Scan 1	268	5′-AGACAAAGAAAGGGAATTCAATC-3′	5′-TGCCATACTAAGTTGTAAACAAATGG-3′
Scan 2	263	5′-CCCTTAGTGTTACACAACAGATCA-3′	5′-AAATTCATGACCTTTTGGACCT-3′
Scan 3	263	5′-TTAGGGAAAATTATGACGGTTTAT-3′	5′-CTTTCATCAGTAACAAAATAATTCAAA-3′
Scan 4	310	5′-TGCTAAATTTGCTTTGACTACTGA-3′	5′-AGCATCACGTCTCCAATAATGA-3′
Scan 5	264	5′-TCTGATTTAGATAAAGGTTTTACCCC-3′	5′-TTGACCACCCATAAGAATACCA-3′
Scan 6	282	5′-GGTGTGAAAATGACTGATCAAGA-3′	5′-TTTTCTAAAAATAGAATGTAATGGCA-3′
Scan 7	274	5′-CCATCAGTCAATAACACTATTAAGGAA-3′	5′-TCCCAAACCCATAATCAACTTC-3′
Scan 8	274	5′-TGCCAAAGCTAATTCTGTTTCA-3′	5′-TTTTTCCCAAATGATTTCTGCT-3′
Scan 9	323	5′-GCCTGACCCTGATTATAGTTCAA-3′	5′-AAATAACCAGTGGACAAAAACCAT-3′

PCR was performed with Go Taq Master Mix (Promega), LCGreen (BioFire Diagnostics) and aprox. 0.2 µg DNA template was filled up with water to reach 15 µl of reaction mixture. HRM reactions were performed using the Bio-Rad CFX96 real-time PCR system. The amplification program was one cycle at 95 °C (2 min) and 95 °C (30 s), 60 °C (30 s), 72 °C (15 s) × 55 cycles followed by: 95 °C for 1 min, and 40 °C for 1 min. Melting of amplicons was conducted by rising the temperature from 67 °C to 91 °C with an 0.2 °C increase step. Melting curve analysis was performed by BioRad Precision Melt Analysis Software and verified visually.

### PCR and DNA Sequencing

Whole *ERG11* gene was amplified as two overlapping parts, each amplified with a unique oligonucleotide primer pair. Gene fragments were then sequenced from both ends with the same primers. The first part primer pair used was as follows: forward primer (5′-AGACAAAGAAAGGGAATTCAATC-3′), reverse primer (5′-TTGACCACCCATAAGAATACCA-3′); the second part forward primer (5′-CGTGATGCTGCTCAAAAGAA-3′), reverse primer (5′-AAATAACCAGTGGACAAAAACCAT-3′). PCR was carried out with Go Taq Master Mix (Promega) and the amplification program was one cycle at 95 °C for 3 min, followed by 45 cycles at 95 °C for 30 s, 58.1 °C for 30 s, and 72 °C for 45 s. Amplicons had the length of approx. 250–300 bp, were verified by agarose gel electrophoresis and were purified with a Wizard SV Gel and PCR Clean-up System (Promega).

DNA reads (Eurofins MWG Operon, Germany; Institute of Medical Biology of PAS, Poland) were screened for the presence of ambiguous nucleotide signals.

## Results

### *Candida albicans* Strains Exhibit Various Resistance to Antifungal Drugs

The susceptibility of reference strains and strains isolated from vagina was determined with microdilution method. MIC was defined as the 50 % inhibition of growth for 5 flucytosine and azole drug tests, and total inhibition of growth for amphotericin B. From among nine *C. albicans* strains tested, one (Ca5V) was classified as resistant to all azole compounds tested (Table 2). Five strains (Ca1V, Ca4V, Ca7V, Ca2V, Ca12r) have shown increased resistance to azole drugs and 5-flucytosine as assessed by drug concentration necessary for complete growth inhibition (data not shown).

**Table 2:** Antifungal drug susceptibility of reference strains and strains isolated from vagina determined with microdilution method

Strain	Susceptibility of reference strains and vaginal isolates, IC50 (mg/l)					
	MIC	KET	ITR	FCA	AMB	5FC
Ca1r	0.0625	0.125	0.25	0.5	0.25	0.25
Ca1V	0.0625	0.125	0.25	0.25	0.5	1
Ca2V	0.0625	0.125	0.25	0.25	0.5	1
Ca3V	0.0625	0.0625	0.25	0.25	0.25	0.125
Ca4V	0.0625	0.25	0.5	0.5	0.5	1
Ca5V	0.5	2	16	64	0.25	1
Ca7V	0.0625	0.125	0.25	0.25	0.25	0.25
Ca11r	0.0625	0.125	0.5	0.5	0.25	0.125
Ca12r	0.0625	0.125	0.5	0.5	0.25	0.5

The growth inhibition of saliva strains was determined visually on ATB FUNGUS3 tests after 24 h incubation (Table 3). Visual readings for most strains did not allow to establish MIC values as they showed very similar growth in all azole drug concentrations. Fourteen strains were classified as resistant (slight decrease in turbidity), and eleven were susceptible (no growth), one strain (Ca21s) was hard to classify (hazy growth). Strain Ca68s showed sharp difference in growth ability as 16 mg/l of fluconazole completely inhibited yeast growth, what is defined as susceptible dose-dependent according to NCCLS interpretive guidelines. Amphotericin B and 5-flucytosine efficiently inhibited growth of all strains.

**Table 3:** Antifungal drug susceptibility of saliva strains determined with ATB FUNGUS3 test

Resistant	Intermediate	Susceptible
*Saliva yeast strain number*		
Ca1s	Ca21s	Ca6s
Ca2s	Ca68s (fluconazole susceptible dose-dependent)	Ca10s
Ca3s		Ca11s
Ca25s		Ca20s
Ca26s		Ca22s
Ca36s		Ca23s
Ca38s		Ca61s
Ca40s		Ca70s
Ca41s		Ca74s
Ca44s		Ca80s
Ca52s		Ca82s
Ca59s		
Ca69s		
Ca81s		

### HRM Analysis Allowed to Distinguish Five Variants of *ERG11* Gene Related to Drug Resistance

HRM analysis was conducted to verify the utility of this method in analysis of *C. albicans ERG11* gene profiles that correlate with drug resistance. Whole sequence of *C. albicans ERG11* gene was analyzed as nine fragments. PCR followed by HRM analysis of products was conducted, which allowed to distinguish between different genetic variants of reference strains and vaginal isolates (Table 4). Initially, one to four variants/clusters of each scan were determined among 9 DNA samples. Then, the same patterns of 9 scans variants/clusters were classified as gene variants and marked 1.–5.

**Table 4:** Variants of scans and *ERG11* gene among reference strains and vaginal isolates identified by HRM analysis

Strain name	Scan number										Gene variants
	1	2	3	4	5	6	7	8	9		
Ca1r	A	A	C	C	A	B	B	C	D	→	3.
Ca1V	A	A	B	B	A	C	A	A	A	→	1.
Ca2V	A	A	A	A	A	A	A	B	B	→	2.
Ca3V	B	A	B′	B	B	B	C	A	C	→	4.
Ca4V	A	A	B	B	A	C	A	A	A	→	1.
Ca5V	A	A	A	A	A	A	A	B	B	→	2.
Ca7V	A	A	B	B	A	C	A	A	A	→	1.
Ca11r	A	A	C	B	A	B	B	C	A	→	5.
Ca12r	A	A	A	A	A	A	A	B	B	→	2.

A, B, C, D—variants of *ERG11* gene fragments among each scan, B′—scan 3 variant not distinguishable by means of HRM, 1–5—whole *ERG11* sequence variants on the basis of scan variants

**Fig. 1:**
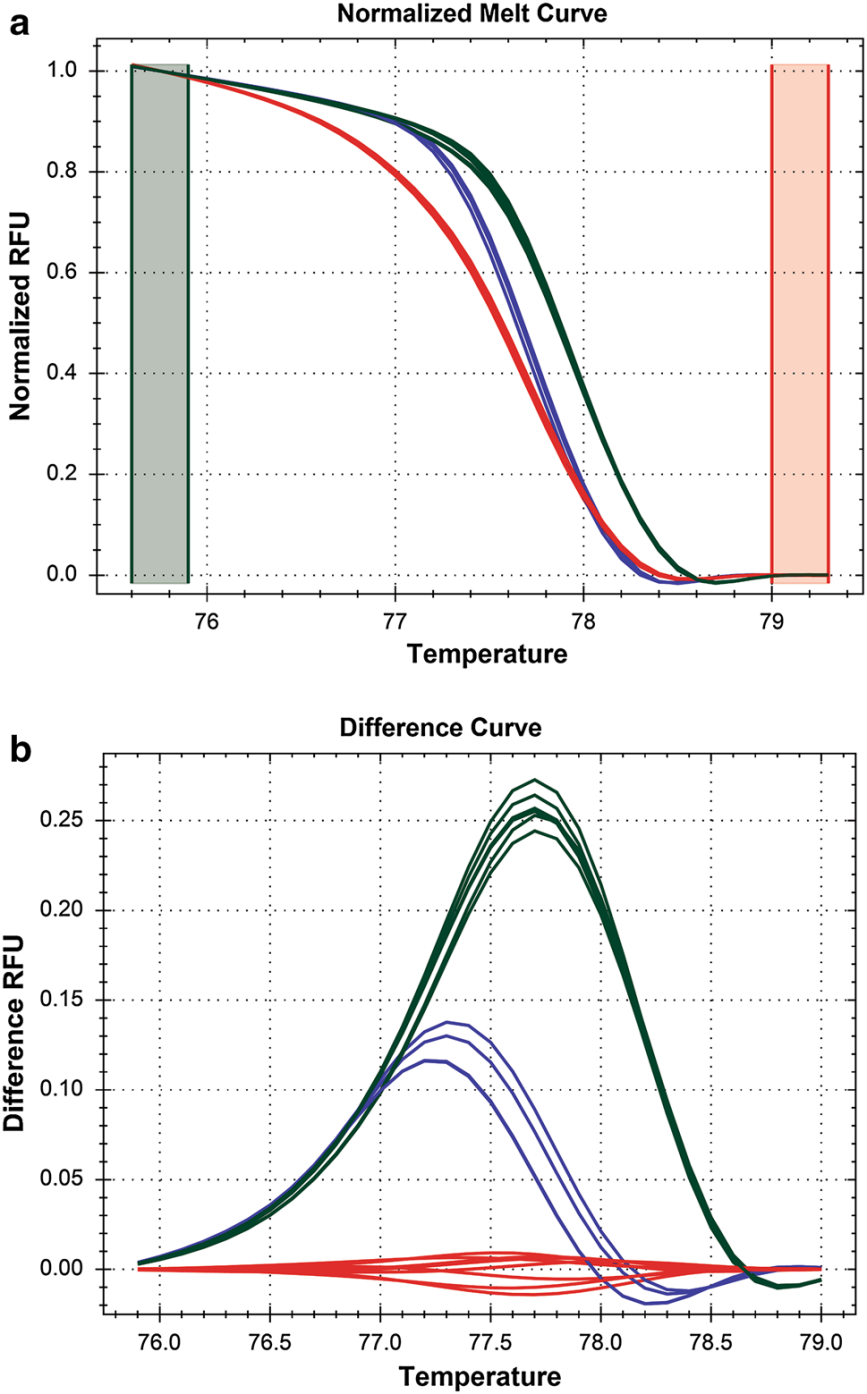
HRM analysis of *ERG11* gene-scan 6 of *Candida albicans* reference strains and vaginal isolated. Both panels (**a** normalized melt curve, **b** difference curve) show three variants of the analyzed gene fragment among nine isolates tested indicating differences in DNA sequences. *Red curves* variant A, *green curves* variant B, *blue curves* variant C (Color figure online)

Isolates Ca1V, Ca4V, Ca7V all were classified as variant 1. as they have the same characteristic HRM profile in scan 3 (variant B) and in scan 6 (variant C), which was unique among 9 isolates tested. Figure 1 shows an example of HRM analysis. Scan 6 of *ERG11* gene was amplified for 9 DNA samples and melting curves of PCR products were analyzed. Normalized melting curve analysis showed three variants of scan 6, which indicates differences in DNA sequence among tested samples (Fig. 1).

Isolates Ca2V, Ca5V, and Ca12r were classified as variant 2. of *ERG11* gene as they represent unique pattern of nine scans variants (Table 4). Here, scan 8 and scan 9 are those, which differentiate the samples from all isolates as they are the only ones showing variant B in both *ERG11* fragments.

Isolates Ca1r, Ca3V, and Ca11r were classified as variant 3., 4. and 5. of *ERG11* gene, respectively, and their patterns of scans variants were unique among isolates tested.

To confirm differences in DNA sequence identified by HRM analysis, all DNA samples were amplified and sequenced. Indeed, isolates Ca1V, Ca4V, Ca7V (variant 1) had the same nucleotide sequence through whole *ERG11* gene. Separate *ERG11* variant was also found for Ca2V, Ca5V, Ca12r isolates (variant 2). Isolates Ca1r, Ca3V and Ca11r represent unique DNA sequence of *ERG11*. Sequence of fragment 3 of Ca3V sample (B′) occurred to be different from this part of Ca1V, Ca4V, Ca7V isolates, but this difference could not be detected by means of HRM. This difference in HRM and sequencing analysis is due to the principle of HRM analysis. The fluorescence signal comes from melting strands depending on their nucleotide content but not on the order of nucleotides. Thus, only by means of sequencing were we able to differentiate variant B (thymine in position 315 to cytosine and cytosine in position 411 to thymine—in strains Ca1V, Ca4V, Ca7V) from variant B′ (cytosine in position 315 to thymine and thymine in position 411 to cytosine—in strain Ca3V).

Regarding the yeast susceptibility to azoles, two gene variants (1 and 2) are related to increased drug resistance, except for strain Ca7V which is susceptible to all tested drugs. Strains numbered Ca1r, Ca3V, and Ca11r, having unique *ERG11* patterns, are the most susceptible ones.

### Sequencing of *ERG11* Gene Revealed Two Point Mutations Associated with *C. albicans* Drug Resistance

Sequencing was the method of choice for analysis of *ERG11* changes in *C. albicans* saliva strains. For *ERG11* sequences of saliva strains the great majority of gene length was successfully sequenced but some fragments of gene were unreadable. The analysis of mutations in *C. albicans* strains of all origins was performed with gPLINK and Haploview software. Drug resistance and susceptibility of strains tested were referred to point mutations in *ERG11* using allelic model. Two intermediate saliva strains (Ca21s, Ca68s) were classified as susceptible for allelic frequency tests. Among 21 point mutations found within ORFs, two were significantly associated with drug resistance (Table 5). Odds ratio based on allelic frequency was calculated in these two cases. Namely, nucleotide substitution at 798 position (Glu266Asp) corresponds to increase of drug resistance. Strains that possess mutated allele variant are 4.7-fold (odds ratio = 4.7143; CI 95 % 1.2368–17.9700) more probable to be drug resistant. In turn, mutation at 1440 position (Leu480Leu) significantly decreases the chance of a strain to be drug resistant (odds ratio = 0.11; CI 95 % 0.0127–0.9504).

**Table 5:** Allele frequencies and Chi-square test results for *C. albicans* resistant (AFF) and susceptible (UNAFF) strains

SNP	A1	A2	Model	AFF	UNAFF	CHISQ test	*P*
UTR_up	2	4	ALLELIC	0/2	2/12	0.3265	0.5677
Phe105Phe	4	2	ALLELIC	14/16	19/21	0.004778	0.9449
Asp116Glu	1	4	ALLELIC	8/22	8/32	0.4321	0.511
Lys119Lys	3	1	ALLELIC	8/22	8/32	0.4321	0.511
Lys128Thr	2	1	ALLELIC	8/22	6/34	1.458	0.2272
Ser137Ser	2	4	ALLELIC	15/15	17/23	0.3886	0.5331
Val159Ile	1	3	ALLELIC	7/23	4/36	2.301	0.1293
His183His	2	4	ALLELIC	9/21	17/23	1.147	0.2841
Le220Leu	2	4	ALLELIC	6/24	11/29	0.5244	0.469
Glu266Asp	2	1	ALLELIC	9/7	6/22	5.495	0.01908*
Val332Val	4	2	ALLELIC	12/12	17/19	0.04449	0.8329
Leu340Leu	3	1	ALLELIC	0/24	2/34	1.379	0.2402
Lys342Lys	3	1	ALLELIC	10/14	11/25	0.7814	0.3767
Ser361Ser	3	1	ALLELIC	0/2	4/10	0.7619	0.3827
Leu370Leu	2	4	ALLELIC	11/13	13/23	0.5671	0.4514
Phe380Phe	2	4	ALLELIC	2/22	4/32	0.1235	0.7253
Tyr401Tyr	2	4	ALLELIC	0/2	6/8	1.371	0.2416
Asp428Asp	2	4	ALLELIC	0/2	4/10	0.7619	0.3827
Ala432Ala	4	2	ALLELIC	14/12	12/24	2.609	0.1063
Ala434Ala	2	4	ALLELIC	14/12	14/22	1.364	0.2429
Leu480Leu	3	1	ALLELIC	1/25	8/22	5.378	0.0204*
Asn490Asn	2	4	ALLELIC	1/25	6/24	3.323	0.06831
UTR_down	2	4	ALLELIC	1/1	6/8	0.03628	0.8489

One missense mutation (Glu266Asp) and one silent mutation (Leu480Leu) statistically significantly correlate with drug resistance (*P* value <0.05, marked *)

*SNP* single-nucleotide polymorphism, *A1* allele 1, *A2* allele2; *1* adenine, *2* cytosine, *3* guanine, *4* thymine

**Fig. 2:**
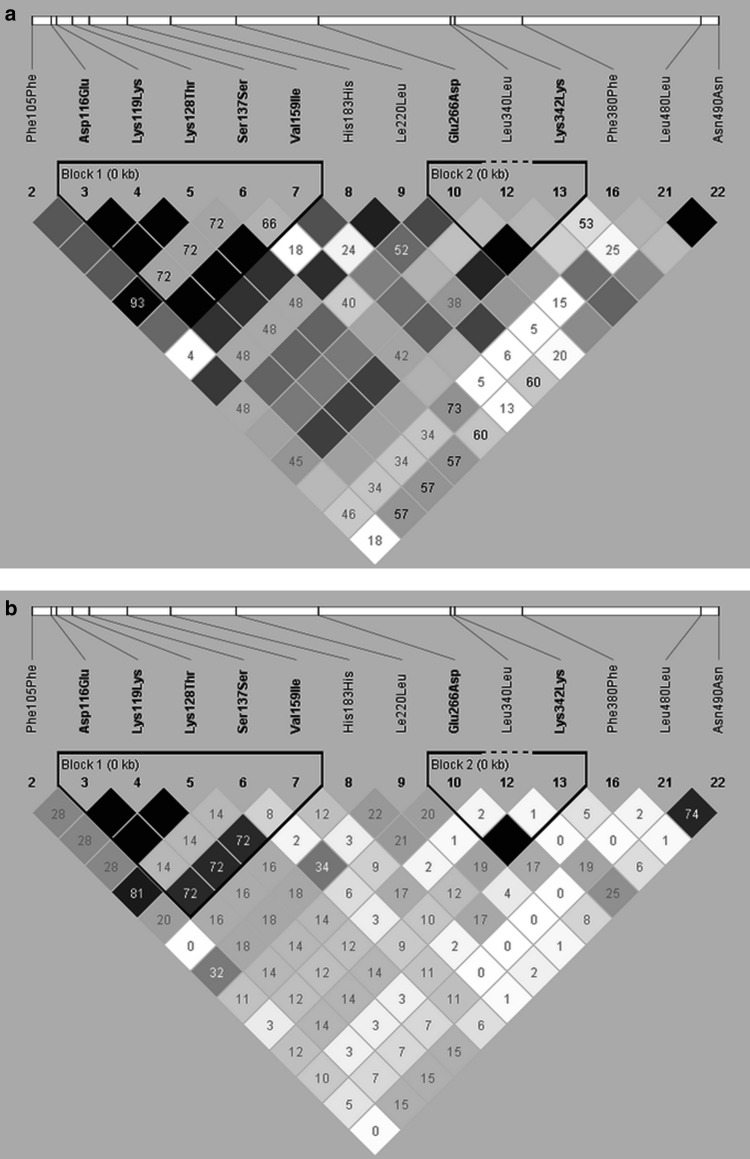
Pairwise linkage disequilibrium (LD) and *r* ^2^ plot. **a** Values on LD plot are *D*′ × 100 and lack of value indicates *D*′ = 1 × 100. *Darker background* indicates greater linkage. **b** Values on *r* ^2^ plot are *r* ^2^ × 100, lack of value indicates *r* ^2^ = 1 × 100. *Denser color* indicates greater linkage (Color figure online)

Pairwise linkage disequilibrium (LD) and *r*^2^ plots were generated on the basis of variants found in *ERG11* gene (Fig. 2a, b). Two blocks of mutations that are inherited together with high frequency were identified. One block of strongly associated mutations comprised Asp116Glu, Lys119Lys, Lys128Thr, Ser137Ser, and Val159Ile. Second block contained Glu266Asp and Lys342Lys.

Sequencing of *ERG11* gene revealed many heterozygous and homozygous mutations but only four of them changed amino acid sequence: D116E, K128T, V159I, E266D (Table 6).

**Table 6:** Amino acid substitutions in clinical and reference strains

Nucleotide position	Amino acid substitutions in *ERG11*	Genotype	Strain name
348 (scan 3)	D116E	TT	Ca1r, Ca1V, Ca3V, Ca4V, Ca11r, Ca2s, Ca3s, Ca6s, Ca10s, Ca20s, Ca21s, Ca23s, Ca26s, Ca38s, Ca40s, Ca41s, Ca44s, Ca68s, Ca74s, Ca80s, Ca81s, Ca82s
		TA	Ca2V, Ca5V, Ca6V, Ca12r, Ca1s, Ca22s, Ca25s, Ca36s, Ca52s, Ca59s, Ca61s
		AA	Ca11s, Ca69s, Ca70s
383 (scan 3)	K128T	AA	Ca1r, Ca1V, Ca3V, Ca4V, Ca11s, Ca2s, Ca3s, Ca6s, Ca10s, Ca20s, Ca21s, Ca23s, Ca26s, Ca38s, Ca40s, Ca41s, Ca44s, Ca68s, Ca70s, Ca74s, Ca80s, Ca81s, Ca82s
		AC	Ca2V, Ca5V, Ca6V, Ca12r, Ca1s, Ca22s, Ca25s, Ca36s, Ca52s, Ca59s, Ca61s
		CC	Ca11r, Ca69s
475 (scan 3)	V159I	GG	Ca1r, Ca1V, Ca2V, Ca3V, Ca4V, Ca5V, Ca11s, Ca12r, Ca2s, Ca3s, Ca6s, Ca10s, Ca20s, Ca21s, Ca23s, Ca26s, Ca38s, Ca40s, Ca41s, Ca44s, Ca68s, Ca70s, Ca74s, Ca80s, Ca81s, Ca82s
		AA	Ca11r, Ca69s, Ca70s
		AG	Ca1s, Ca22s, Ca25s, Ca36s, Ca52s, Ca59s, Ca61s
798 (scan 5)	E266D	AA	Ca1r, Ca1V, Ca2V, Ca4V, Ca5V, Ca11r, Ca12r, Ca10s, Ca11s, Ca22s, Ca52s, Ca61s, Ca69s
		CC	Ca3V, Ca3s, Ca23s, Ca40s, Ca41s, Ca44s
		AC	Ca74s, Ca80s, Ca81s

## Discussion

Various mechanisms of drug resistance in *C. albicans* have been studied in recent years. Various authors agree that many different phenomena occurring simultaneously in a yeast cell are responsible for gaining drug resistance by *C. albicans* strains [7, 33, 40]. Mechanisms based on overexpression or hyperactivity of CDR1, CDR2, and MDR1 efflux pumps have been widely investigated [2, 19, 27]. Promising, but not yet very common are other approaches to the problem concerning mutations in CDR1 and CDR2 transcription factors [3], specific chromosome alterations [37], and mutations in various ergosterol synthesis pathway genes, like ERG5 or ERG3 [22]. Moreover, many genetic studies indicate that mutations in *ERG11* or overexpression of these azole drugs target gene cause increased resistance to commonly administered antifungal drugs [14, 31, 43].

In this study, we present the application of HRM method to discriminate different variants of *ERG11* gene in *C. albicans* strains on the basis of single-nucleotide polymorphisms. Among nine strains tested, three isolates Ca1V, Ca4V, Ca7V had the same variant of *ERG11* gene sequence. Characteristic pattern is composed by mutations present in scan 3 and scan 6. Another distinctive sequence was determined by HRM analysis for Ca2V, Ca5V, and Ca12r isolates which represent variant 2 and their *ERG11* gene sequence differs from other isolates in scan 8 and 9. Three other variants of *ERG11* gene sequence were unique among strains tested. Sequence differences between variants were confirmed by sequencing reaction for all isolates included in HRM analysis.

These results indicate that HRM analysis could be a useful tool for classifying *C. albicans* isolates to distinctive variants of *ERG11* gene. Similar approach has been described by Strzelczyk who used MSSCP method for preliminary selection of isolates presenting differences in *ERG11* gene, prior to sequence analysis [36]. Rapid detection of *ERG11* mutations was also performed by Loeffler et. [16] who used fluorescence resonance energy transfer and probed melting curves for this purpose. Another approach to determine *ERG11* mutations consisted in the use of padlock probe and rolling circle amplification (RCA)-based method [39]. As the alternative to usual DNA sequencing, bioluminometric pyrosequencing method was employed to detect *ERG11* mutations [10].

Considering low complexity of HRM reaction preparation and high resolution of analysis results, we propose HRM analysis as an alternative method of sample selection for subsequent sequencing. Nota bene, multi-well plate format is used for HRM reaction, which gives the possibility to predict mutations in tested sample using a panel of *ERG11* gene variants for HRM analysis prepared in advance. Thus, HRM analysis should be seriously considered in attempts to accelerate diagnosis of *Candida* infection.

Drug susceptibility tests revealed that one out of nine strains was highly resistant to antifungal drugs (Ca5V) and was also resistant to itraconazole and fluconazole according to NCCLS breakpoints [26]. Five other strains presented increased resistance to at least one drug (Ca1V, Ca4V, Ca2V, Ca11r, and Ca12r) and only three of them were resistant to an azole drug. Namely, they were resistant to itraconazole with MIC = 0.5 mg mL^−1^, which is classified as dose-dependent susceptibility (S-DD) according to NCCLS.

In general, resistant strains represented variant 1. or variant 2. of *ERG11* gene sequence with the exception of strain Ca11r, which was classified as distinct variant by HRM and by sequencing. It suggests the existence of another drug-resistance mechanism which causes slightly increased resistance to itraconazole of Ca11r strain. It is well documented that *C. albicans* strains develop many different mechanisms of resistance to azole drugs simultaneously [7, 30]. Some authors also suggest that due to the complexity of resistance in *C. albicans* it is necessary to analyze groups of resistant and susceptible strains that are matched, in order to reliably identify the mechanism responsible for azole resistance [40]. Moreover, heterogeneous mechanisms of resistance may develop within clonal origin subpopulations under azole exposure [23]. On the other hand, several studies indicate that some *ERG11* point mutations may occur in azole susceptible, as well as in resistant strains [21, 30, 40]. This is in line with our observation in isolate Ca1V, Ca2V, and Ca7V that do not show increased resistance to any azole drug, although being members of variants 1 or 2. of *ERG11*, which we initially considered characteristic for drug-resistant strains.

Thus, in order to correlate *C. albicans* drug resistance with *ERG11* gene variants using HRM method, it seems to be important to primarily recognize single-nucleotide polymorphism, which is related to increased resistance. Then, it could be possible to design more exact HRM tests with short fragments of *ERG11* gene embracing statistically significant point mutations in *ERG11* sequence. For this purpose, a numerous group of *C. albicans* isolates are needed. Here, we present *ERG11* sequencing results for the collection of 27 *C. albicans* strains isolated from saliva of healthy volunteers. The statistical analysis was performed for strains of all origins. Among them, 21 nucleotide substitutions were recognized, and two of them were associated with changes in drug resistance with statistical significance (Chi square test, *P* < 0.05). Strains that carry substitution at position 798 (Glu266Asp) for at least one allele are 4.7-fold more probable to be azole resistant. This is in agreement with the results of Loffler who identified Glu266Asp substitution in 5 out of 19 resistant strains [17]. None of 19 susceptible strains showed this mutation. However, many authors suggest that substitution is not associated with resistance as it occurs in resistant, as well as in susceptible strains [24, 40]. Glu266Asp amino acid substitution was also shown not to be related to decreased affinity of fluconazole to *ERG11* gene product, cytochrome P-450 [18]. Another substitution, which had statistical significance, was silent mutation at position 1440 (Leu480Leu) which statistically decreases probability of resistance in *C. albicans* strains. Nucleotide substitution at this site was also found by others [22, 23]; however, to the best of our knowledge no one have ever estimated its significance in drug resistance prediction. The impact of silent polymorphisms has been considered as a risk factor in disease development of higher eukaryotic organisms [34]. Silent mutations may cause structural and functional changes in synthesized protein [12]. Many mechanisms of this phenomena were proposed, one of which is change in translation kinetics and altered protein folding [13]. Other explanation may be that silent mutations cause native splicing donor site inactivation [11]. This change would shorten mRNA chain length of *ERG11* and the usage of RT-PCR could confirm this hypothesis in our study. It is also possible that alternate splicing caused changed protein or small RNA binding or synonymous SNPs changed mRNA stability what consequently led to lower lanosterol demethylase expression and decrease of yeast drug resistance [32]. Also a polar effect to other gene can be considered, although it has only been shown for *Saccharomyces cerevisiae* mutants [9]. Taking the above into account, we suggest that silent mutations, frequently occurring in *ERG11* gene, should also be considered as drug-resistance markers when establishing new genetic prediction methods.

On the basis of the presented data, HRM analysis seems to be a useful screening method that allows to select DNA samples for subsequent sequencing of *ERG11* gene, thus cheapening and shortening the process of *Candida* infection diagnosis. However, it is not a reliable method of classification of *C. albicans* resistant and susceptible strains, firstly because other drug-resistance mechanisms may occur simultaneously, and secondly because our knowledge of changes in *ERG11* responsible for azole resistance is not yet sufficient to design proper and trustworthy test based on *ERG11* mutation sites. Nevertheless, the number of current studies on *C. albicans* drug resistance increases, and they lead to better understanding of the significance of *ERG11* mutations, which makes scanning methods like HRM analysis a promising tool for the future diagnosis of *C. albicans* infections.
